# The use of cone-beam computed tomography (CBCT) in radiocarpal fractures: a diagnostic test accuracy meta-analysis

**DOI:** 10.1007/s00256-021-03883-9

**Published:** 2021-09-20

**Authors:** Emma Fitzpatrick, Vivek Sharma, Djamila Rojoa, Firas Raheman, Harvinder Singh

**Affiliations:** 1 Department of Trauma and Orthopaedics, Leicester Royal Infirmary, University Hospitals of Leicester, Leicester, UK; 2 Department of Plastics Surgery, Leicester Royal Infirmary, University Hospital of Leicester, Leicester, UK

**Keywords:** Cone-beam computed tomography, CBCT, Scaphoid fracture, Carpal fracture, Distal radius fracture

## Abstract

**Objective:**

Occult radiocarpal fractures often present a diagnostic challenge to the emergency department. Accurate diagnosis of these injuries is crucial as a missed fracture can lead to significant morbidity. Cone-beam CT (CBCT) scan is a novel imaging modality, with minimal radiation exposure and comparatively fast acquisition time. Our aim was to evaluate its use in the diagnosis of cortical fractures in the upper limb extremity.

**Materials and methods:**

We conducted a systematic review of literature and included all studies that evaluated the use of CBCT in the diagnosis of radiocarpal fractures. We used a mixed-effects logistic regression bivariate model to estimate the summary sensitivity and specificity and constructed hierarchical summary receiver operative characteristic curves (HSROC).

**Results:**

We identified 5 studies, with 439 patients, and observed CBCT to be 87.7% (95% CI 77.6–93.6) sensitive and 99.2% (95% CI 92.6–99.9) specific for scaphoid fractures. For carpal fractures, CBCT was observed to have a pooled sensitivity and specificity of 90.6% (95% CI 72.7–97.2) and 100% (95% CI 99–100) respectively. For distal radius fractures, CBCT sensitivity was 90% (95% CI 67–98) and specificity was 100% (95% CI 10–100). The overall inter-rater agreement effect was shown to be 0.89 (95% CI 0.82–0.96), which is deemed to be almost perfect.

**Conclusion:**

CBCT is an accurate diagnostic tool for occult radiocarpal cortical fractures, which could replace or supplement radiographs. We believe CBCT has a promising role in the acute radiocarpal fracture diagnostic algorithm in both emergency and trauma departments.

## Introduction

Carpal fractures account for 8% of all hand fractures of which scaphoids are the most common [1]. Early diagnosis of these fractures is important as delay may result in considerable morbidity arising from complications such as non-union, carpal instability, osteoarthritis or avascular necrosis [2].

Radiographs are the initial imaging modality of choice but display a relatively poor diagnostic accuracy with a sensitivity between 66 and 81% for scaphoid fractures, 39% for carpal fractures overall, and 58% for wrist fractures [3, 4]. Cross-sectional imaging provides a higher diagnostic yield. For scaphoid fractures, conventional computerized tomography (CT) has a sensitivity and specificity of 82% and 96% respectively, whilst magnetic resonance imaging (MRI), considered the gold standard, has the highest sensitivity and specificity of 94% and 98% respectively [5].

Carpal fractures are often radiographically occult, with up to 30% of fractures missed on radiographs [4]. Multiple factors contribute to their difficult detection including overlapping structures, poor technique, suboptimal positioning and lack of dedicated special radiographic views. Traditionally, unlike for other carpal bones, patients with radiographically occult scaphoid fractures are immobilized in a cast for two weeks followed by repeated clinical and radiological examination. This old dictum results in significant overtreatment with eventually only 4–20% of patients being diagnosed with scaphoid fractures [6]. Therefore, most protocols now recommend early MRI or CT for suspected scaphoid fractures [7]. Despite this, there is significant heterogeneity in imaging techniques employed in UK centres—only 51% of respondents to a recent survey offering MRI to patients within 2 weeks, with accessibility and cost-implications commonly reported limitations [8].

**Fig. 1:**
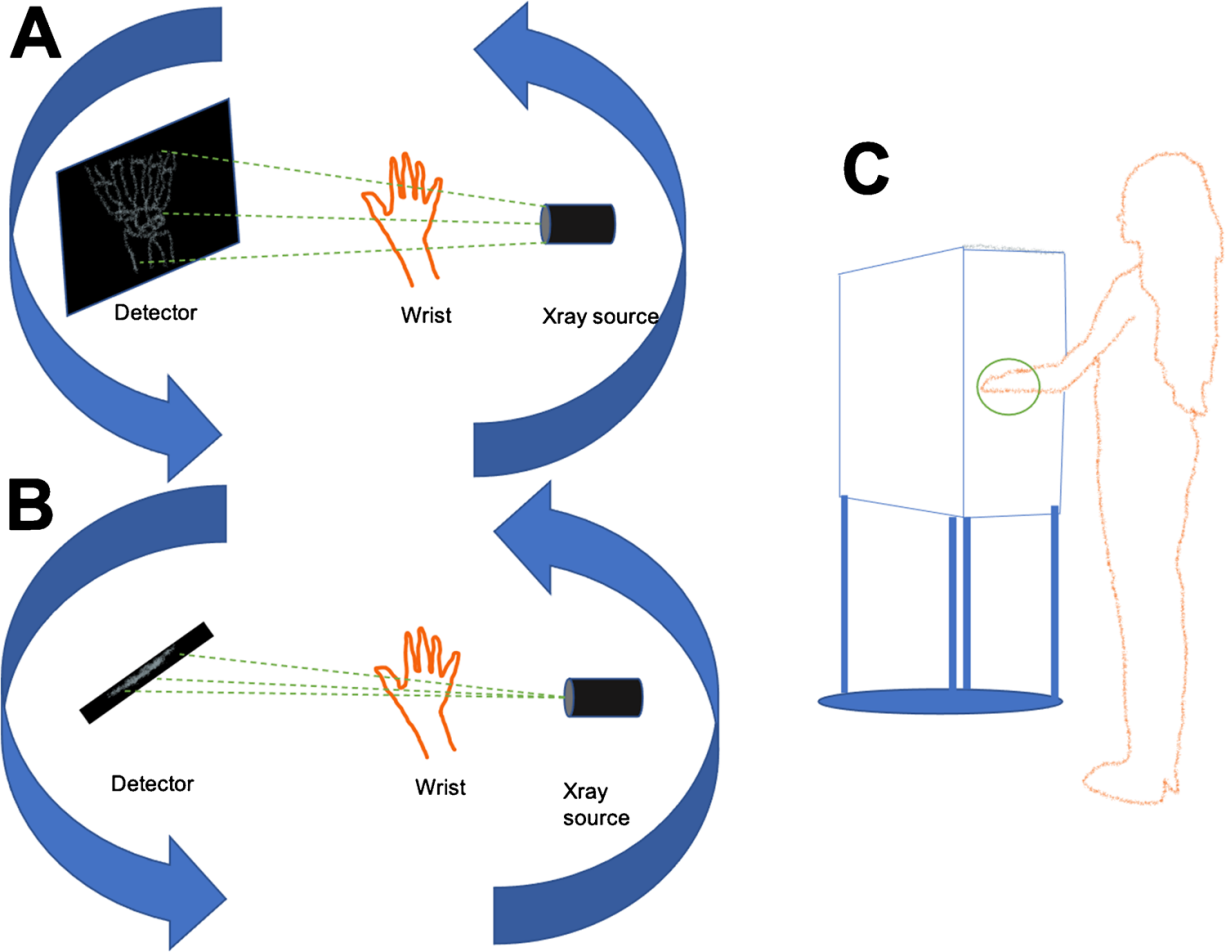
**a**–**c** Schematic illustrating the differences in image acquisition of CBCT compared to conventional CT. **a** CBCT utilizes a cone-shaped beam recorded by a flat-panel detector, requiring only a single rotation around an object. **b** Conventional CT device utilizes an X-ray beam is a fan-shape and is detected by an arc shaped panel rotating in a direction opposite to the X-ray source. **c** Position of a patient during CBCT performed of the wrist

Cone-beam CT (CBCT), utilized extensively in the field of dentistry since the 1990s, is increasingly being investigated for the evaluation of radiocarpal fractures. CBCT uses a large, flat area detector and a cone-shaped X-ray beam to acquire volumetric data from multiple projections in a single rotation around an object (Fig. 1) [9]. Compared to conventional multidetector CT (MDCT), CBCT produces high spatial resolution images at a low radiation exposure, further reduced by scanner design which allows shielding of radiosensitive organs [10].

CBCT has been shown to perform significantly better than radiography in the detection of small bone and joint fractures and may have a diagnostic value at least similar to MDCT [11]. As CBCT is fast to perform and likely has a higher diagnostic yield than radiography, it may have particular utility for the diagnosis of radiographically occult extremity fractures in the acute trauma setting [12, 13]. However, the accuracy and efficacy of CBCT are yet to be proven for radio-carpal fractures. The objective of this study is to investigate the accuracy of CBCT in the diagnosis of radiocarpal fractures.

## Methodology

### Design

A study protocol set out the objectives of the review, study inclusion criteria and methods of analysis and was prospectively registered with PROSPERO (CRD42021224232). The review was reported in accordance with Preferred Reporting Items for Systematic Review and Meta-analysis (PRISMA) of diagnostic test accuracy studies.

### Criteria for considering studies for this review

Retrospective observational, cohort or cross-sectional and prospective studies that assessed the use of CBCT in the diagnosis of scaphoid fractures were included. Case studies and reports of less than five patients, review articles, conference abstracts, meta-analysis and commentaries were excluded. As CBCT is an emerging imaging modality for upper limb extremity injuries, included studies could not be restricted to randomised controlled trials due to lack of available data.

### Participants

All participants over the age of 18 who presented with acute traumatic carpal or distal radius injuries which were imaged using CBCT were included. Bony injuries included distal radius, carpal, and scaphoid fractures, which were managed surgically or conservatively with clinical follow-up.

### Target condition

Radiocarpal fractures were defined as fractures of the distal radius and any carpal bone. Carpal bone fractures were defined as fractures of any carpal bones, including the scaphoid. We also included injuries where there was ongoing clinical suspicion of scaphoid and other radiocarpal cortical fractures, despite an initial negative radiograph. Injuries with negative initial radiographs which had ongoing clinical suspicion of injury were also included.

### Index test

CBCT uses a cone-shaped X-ray beam and 2-D detectors with multiple pixels in the *x* and *y* axis, instead of a one-dimensional detector and a fan-shaped X-ray beam as for a fan-beam CT detector [14, 15]. Compared to conventional CT, cone-beam CT (CBCT) has a higher resolution, 90% lower radiation and a faster scanning time [14]. It thus reduces examination time and provides less movement artefacts. However, it requires an appropriate reconstruction algorithm and increases scattered radiation. Recent studies have demonstrated CBCT may have similar accuracy in detecting occult fractures of the wrist and scaphoid bone as MRI [5, 15, 16].

### Reference standards

Plain radiographs are the standard initial imaging modality for suspected radiocarpal injuries, usually including posteroanterior (PA), true lateral, oblique, and scaphoid views of the wrist. All patients included in the study underwent radiographs and CBCT. MRI scan is the gold standard imaging modality for radiocarpal fractures and was used on its own or in combination with clinical follow-up and surgical findings as a reference standard [10, 15, 17–19].

### Search methods for identification of studies

The literature search was undertaken in December 2020 over a 3-week period by two review authors (EF and VS). The Healthcare Databases Advanced Search (HDAS) interface by the National Institute for Health and Care Excellence (NICE) was used to run searches in the PubMed/Medline/EMBASE databases. A list of search strategy keywords was compiled and used to search the literature (Supplementary material). Only studies in English were included.

### Data collection and analysis

#### Selection of studies

**Fig. 2:**
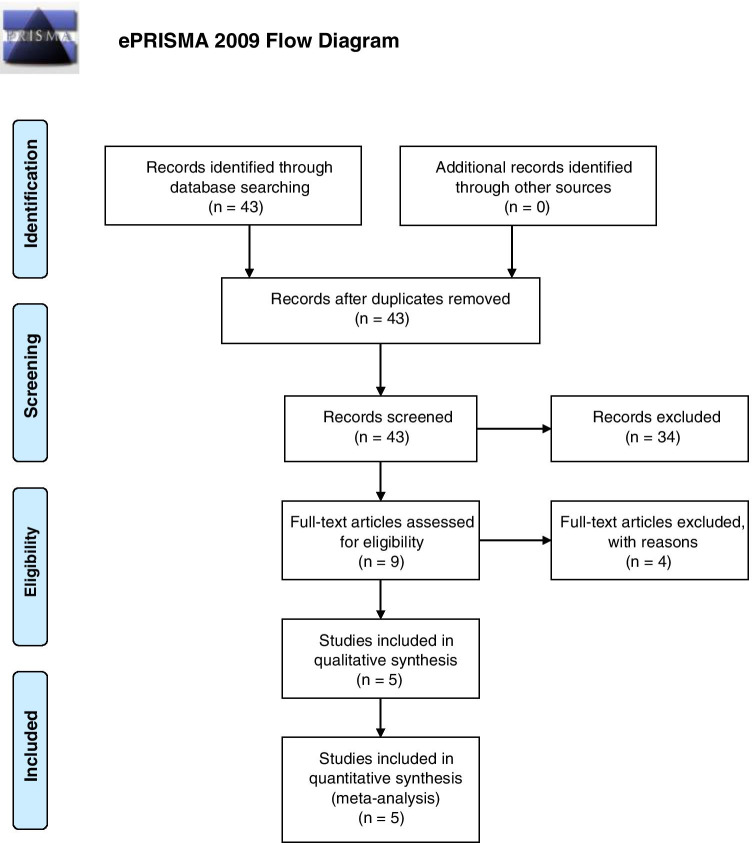
The PRISMA diagram for the search strategy

Eligible studies were assessed by two review authors (EF, VS) independently. A third author (DMR) acted as an arbitrator when consensus could not be reached between the first two. Titles and abstracts of the electronic database search results were first screened, as shown in the PRISMA diagram in Fig. 2. Full-text articles of the studies meeting the included criteria were then reviewed.

#### Data extraction

Data was retrieved by one review author (EF) and cross-checked by a second author (VS). The following data were extracted from the full-text articles of the selected studies:

- Article (author, year and journal of publication)
- Study design (sample size, type of study)
- Study population and demographics (age, gender)
- Reference standard (clinical follow-up, MRI scans, intraoperative findings)
- Index test (CBCT) and its interpretations
- Quality assessment of the included studies using the Quality Assessment of Diagnostics
- Accuracy Studies (QUADAS-2) tool [20]
- Absolute number of true positives, true negatives, false positives, false negatives, sensitivity, specificity, accuracy for two-by-two contingency tables

#### Assessment of methodological quality

The methodology of the selected studies was assessed using the revised QUADAS-2 tool on RevMan 5.3 (Cochrane Collaboration, Copenhagen, Denmark). The four domains used to assess the risk of bias were participant selection, index test, reference standard and flow and timing. Applicability was assessed for the first three domains, within each domain the questions were answered with “yes”, “no” or “unclear” and the risk of bias was classified as “low”, “high” or “unclear”. The methodological quality assessment was performed by two review authors (EF, VS) independently.

#### Statistical analysis and data synthesis

Dichotomous outcomes for both the reference standard and index test were obtained from the included studies. Two by two contingency tables were constructed and classified according to the reported true positives, true negatives, false positives and false negatives. Sensitivity and specificity with 95% CI were calculated for each study using RevMan 5.3 and presented through forest plots. A mixed-effects logistic regression bivariate model was used to meta-analyse each index test interpretation individually, where logit transformed sensitivities and specificities were modified. Summary receiver operating characteristic (SROC) curves were constructed and summary sensitivities and specificities were computed. Through this, we obtained diagnostic odds ratio (DOR) and positive (LR+) and negative (LR−) likelihood ratios. Hierarchical modelling was performed to generate hierarchical summary ROC (HSROC) curves to present summary points, 95% confidence regions and 95% prediction regions. Studies reporting inter-observer agreement for index test interpretation were meta-analysed using a mixed-effects model through calculated standard errors from the reported agreement coefficients [21]. All analysis was undertaken using “metan”, “midas” and “metandi” commands in Stata 16 (StataCorp, College Station, TX, USA).

#### Assessment of reporting bias

We have only included 5 studies, which is not a sufficient number for reporting bias using a funnel plot [22].

## Results

### Results of search

The literature search performed on the Healthcare Databases Advanced Search (HDAS), including Medline, PubMed and EMBASE, for “scaphoid”, “hand/s”, “carpal/s”, “distal radius” or “distal radial” produced 626,704 results. “Injury/s”, “trauma” or “fracture/s” produced 1,031,290 results. “Cone beam computed tomography”, “cone beam CT”, “CBCT” or “CB-CT” produced 14,092 results. A combination of all three searches obtained 43 results. The title and abstract screen of the 43 results produced nine papers. Two papers used the same data; the paper which focused on scaphoid fracture was included [12, 23]. One paper did not distinguish between the results of upper and lower limb fractures and was therefore excluded [24]. The other two studies were irrelevant and therefore not included [11, 25]. Five studies were included in the paper [10, 15, 17–19].

### Characteristics of studies

The characteristics of all studies are summarised in Table 1. Only acute traumatic injuries were included. Three studies prospectively recruited patients and two of these studies included patients with clinical suspicion of scaphoid fracture in whom the initial X-ray was negative. The third included all patients with a suspected scaphoid fracture [17]. Two studies retrospectively identified cases, one included all trauma to distal upper and lower limbs whilst the other included clinical suspicion of scaphoid fracture only, and all included patients underwent X-ray and CBCT at time of presentation [10, 26]. The mean age ranged from 33 to 49. The total number of patients included was 439, with 381 proven to have fractures by CBCT. For all studies, imaging was interpreted by at least one radiologist. In three studies, the radiologists were blinded to all previous imaging and the other radiologist’s report [15, 18, 19]. In the other two studies, the interpreting radiologist worked in consensus [10, 17]. Four of the studies also blinded the radiologist to the clinical information [10, 17–19]. In addition to a radiologist, an orthopaedic consultant also reviewed the images in two studies [10, 19]. One study used MRI as the reference standard for all patients [18]. The other four studies used radiographs and CBCT for all patients with follow-up clinical examination and additional imaging (MRI) used as the reference standard. Table 2 shows the characteristics of different imaging modalities used.

**Table 1:** Characteristics of all included studies

Author	Year	Type of study	Inclusion criteria	Injury to image time	Mean age	Patients	DUL	S	C	DR	Interpretation	Reference standard
Borel et al.	2017	P	Traumatic clinical suspicion of scaphoid fracture with normal X-ray	7 days, X-ray, CBCT, MRI done within 7 days	36	49	24	16	18	4	1 senior and 1 junior radiologist	MRI (cortical fracture identified by hypo T1 signal, trabecular by hyper-Dixon signal and hypo-T1 signal)
Edlund et al.	2015	P	Clinical suspicion of scaphoid fracture within 7 days of trauma	7 days, CBCT within 24 h of X-ray	40	95	45	11	32	11	2 senior radiologist	Clinical follow-up and additional imaging (MRI)
Gibney et al.	2019	P	Acute traumatic clinical suspicion of radiocarpal fractures with normal X-ray	14 days, of injury and X-ray	41	117	61	9	44	13	1 senior and 1 junior radiologist	Clinical follow-up and additional imaging (MRI)
Grunz et al.	2020	R	Acute traumatic injury to distal upper and lower limb	Not specified, X-ray and CBCT same day	49	76	54	8	15	39	1 senior and 1 junior radiologist	Surgical reports, clinical follow-up and additional imaging (MRI)
Neubauer et al.	2018	R	Traumatic, clinical suspicion of scaphoid fracture	Not specified, X-ray and CBCT within 4 days	33	102	NA	197	NA	NA	4 radiologists	Clinical follow-up and additional imaging (MRI)

*P* prospective, *R* retrospective, *DUL* distal upper limb fracture, *S* scaphoid fracture, *C* carpal fracture, *DR* distal radial fracture

**Table 2:** Characteristics of different imaging modalities used by included studies

Author	Year	Radiographs	CBCT	MRI
Borel et al.	2017	•4 views: AP, lateral, ulnar deviation (× 2) •Unit:ProGrade Eleva, Philips Medical Systems, Eindhoven, Netherlands •Protocol: 50 kV & 4 mA	•Unit: Planmeca ProMax 3D mid, Helsinki, Finland •Protocol: 90 kV & 120 mA •Acquistion time: 15 s •Field: 90 × 90mm •Slice thickness: 0.5 mm	•Unit: 3-T unit, Magnetom Skyra, Siemens Healthcare •Protocol: Dixon & T1 spin-echo •Field: 100 × 100 mm •Slice thickness: 2.5 mm
Edlund et al.	2015	•4 views •Units: a Mediel Classic, Kodak DR 7500, Carestream DRX Evolution	•Unit: Planmed Oy, Helsinki, Finland •Protocol: 6 mA & 90 kV,12 mA & 80 kV, 12 mA and 96 kV •Acquistion time: 36 s •Field: 160 × 130 mm •Slice thickness: 0.2 mm	•Units: 1.5-T GE Optima, 1.5-T GE Optima HDxt Edition 23 •Protocol: localizer, coronal short tau inversion recovery (STIR) sequence, coronal sequence, sagittal T1 sequence •Acquisition time: 10 min •Field: 140 × 150 mm •Slice thickness: 3 mm
Gibney et al.	2019	•4 views: PA, lateral, obliqe, scaphoid PA •Unit: Discovery XR656 •Protocol: 55 kV & 3 mA	•Unit: planmed verity •Protocol: 90 kV & 6 mA •Acquistion time: 30 s •Field:150 mm × 150 mm •Slice thickness: 0.2 mm	•3 T Magnetom Skyra •Protocol: coronal slices T1-weighted spin-echo, double-echo steady-state •Field: 100 × 100 mm •Slice thickness: 2.4 mm & 0.4 mm
Grunz et al.	2020	•4 views •Unit: twin robotic xray system (Multitom Rax, Siemens Healthineers) •Protocol: 50–55 kV, 2–2.2 mA	•Unit: twin robotic X-ray system (Multitom Rax, Siemens Healthineers) •Protocol: 78.8 kV, 114.7 mA •Acquistion time: 20 s •Field: 80 × 80 mm •Slice thickness: 2 mm	
Neubauer et al.	2018	•4 views •Protocol: 55 kV	•Unit: verity; Planmed •Protocol: 90 kV, 36 mA •Slice thickness: 0.2 mm	

### Methodological quality of included studies

**Fig. 3:**
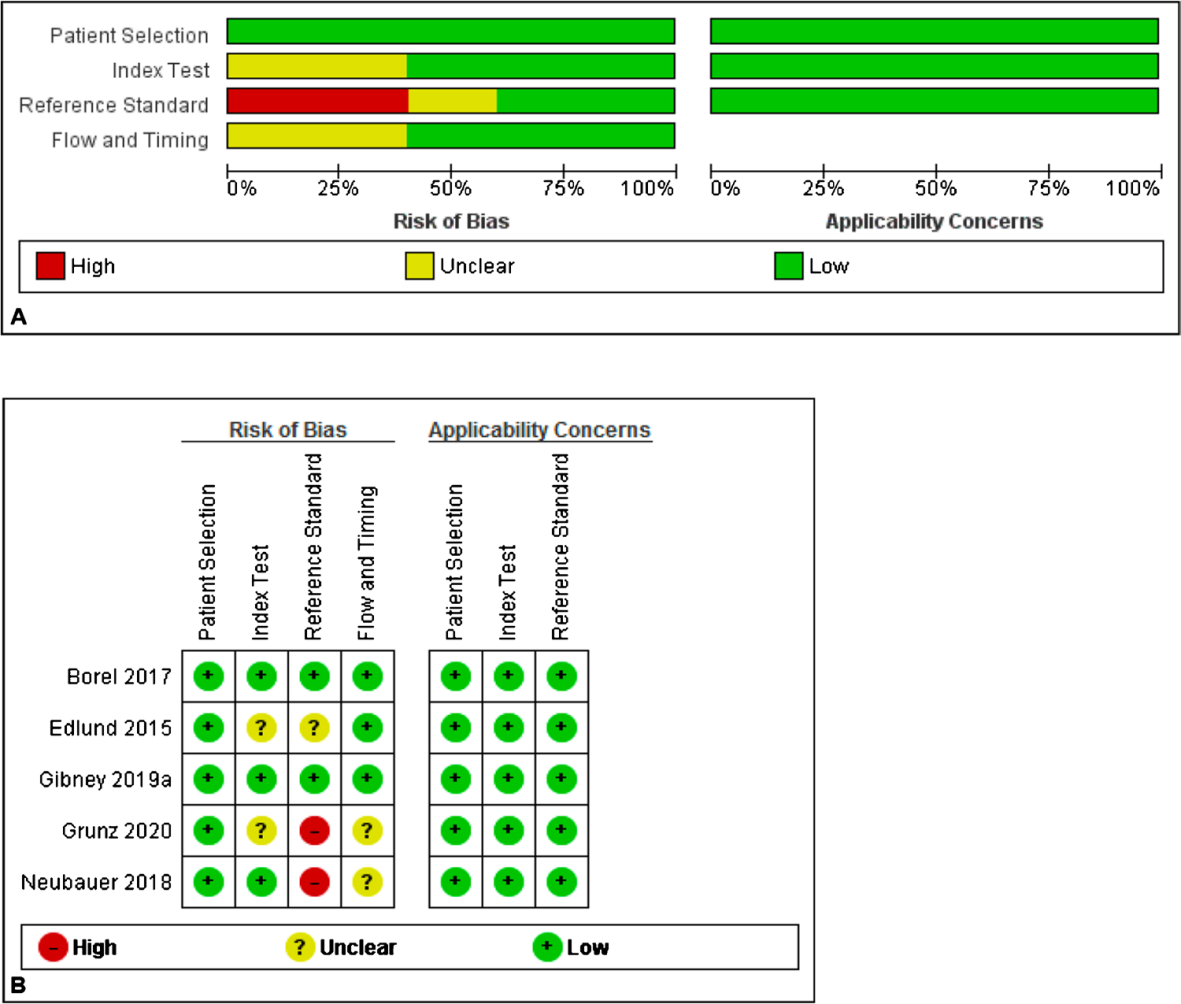
**a** The methodological quality assessment summary. **b** The methodological assessment for each individual study included

The risk of bias analysis is summarised in Fig. 3a and b. Only one study used MRI for all patients as the reference standard [18]. The other four studies used clinical follow-ups with or without MRI as the reference standard. Differential reference bias can reduce the accuracy of the study findings. The majority of studies used blinded radiologists to interpret the imaging results; however, in Grunz et al. [10], the interpreting radiologists were aware of the original imaging results. Gibney et al. [15] categorized radiographically occult fractures based on initial radiology reports. They retrospectively found that if all such radiographs were reviewed by an experienced musculoskeletal radiologist, 17% of initially labelled ‘occult’ fractures would have been identified. However, there is limited feasibility of musculoskeletal radiologists reporting all acute injuries in the emergency setting [15]. Studies which used first-visit follow-up to select patients may be prone to selection bias as patients who had resolving pain may not have attended follow-up. The study by Edlund et al. [17] was subject to attrition bias with 15% of patients not undergoing scheduled follow-up MRI. Moreover, MRI was carried out 2 weeks after CBCT scans.

### Findings

**Fig. 4:**
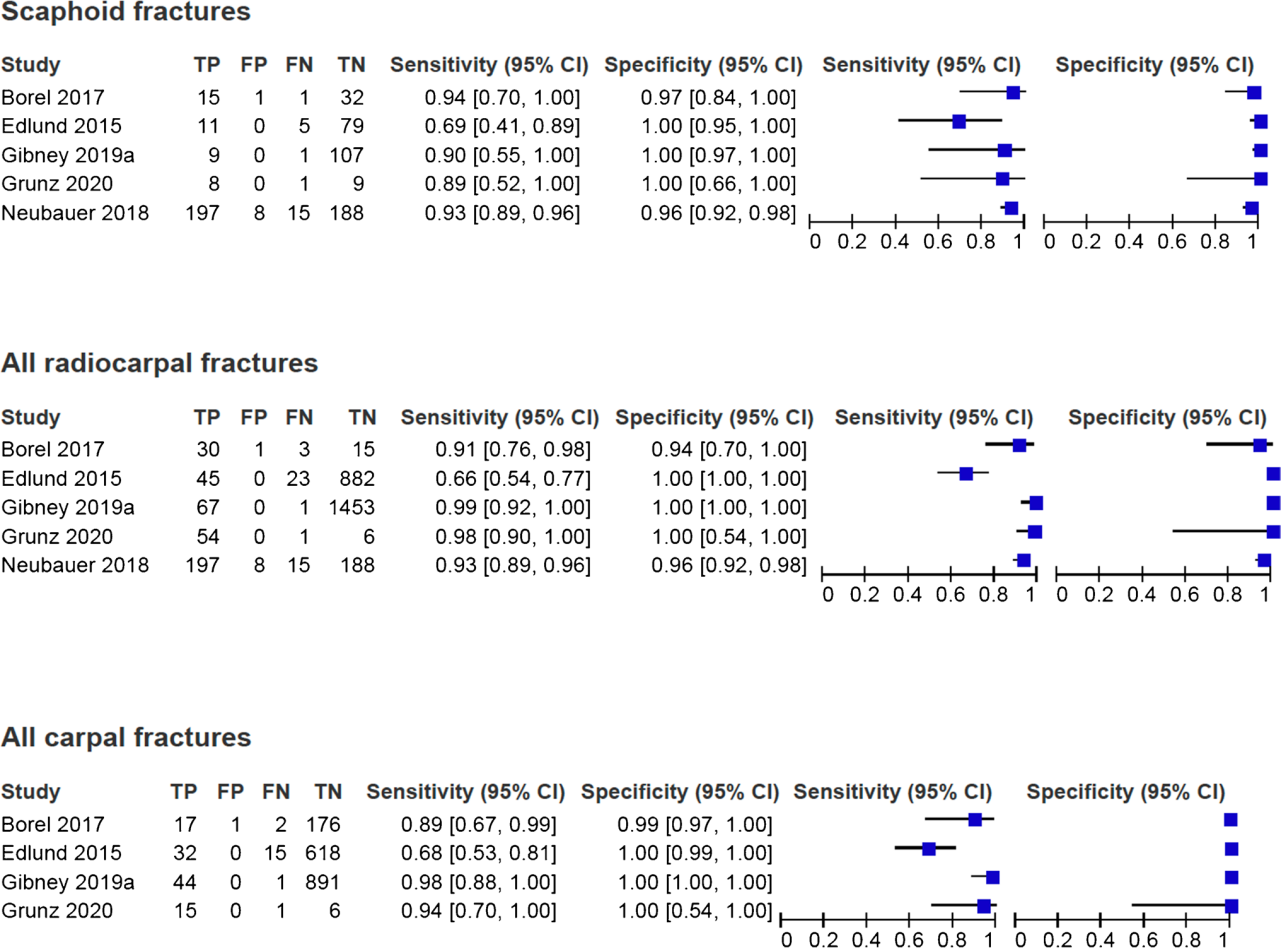
The forest plots for sensitivity and specificity for CBCT identifying radiocarpal fractures

The forest plots for sensitivity and specificity for CBCT identifying radiocarpal fractures are displayed in Fig. 4 with a breakdown for scaphoid fractures and all carpal fractures individually.

#### Scaphoid fractures

**Fig. 5:**
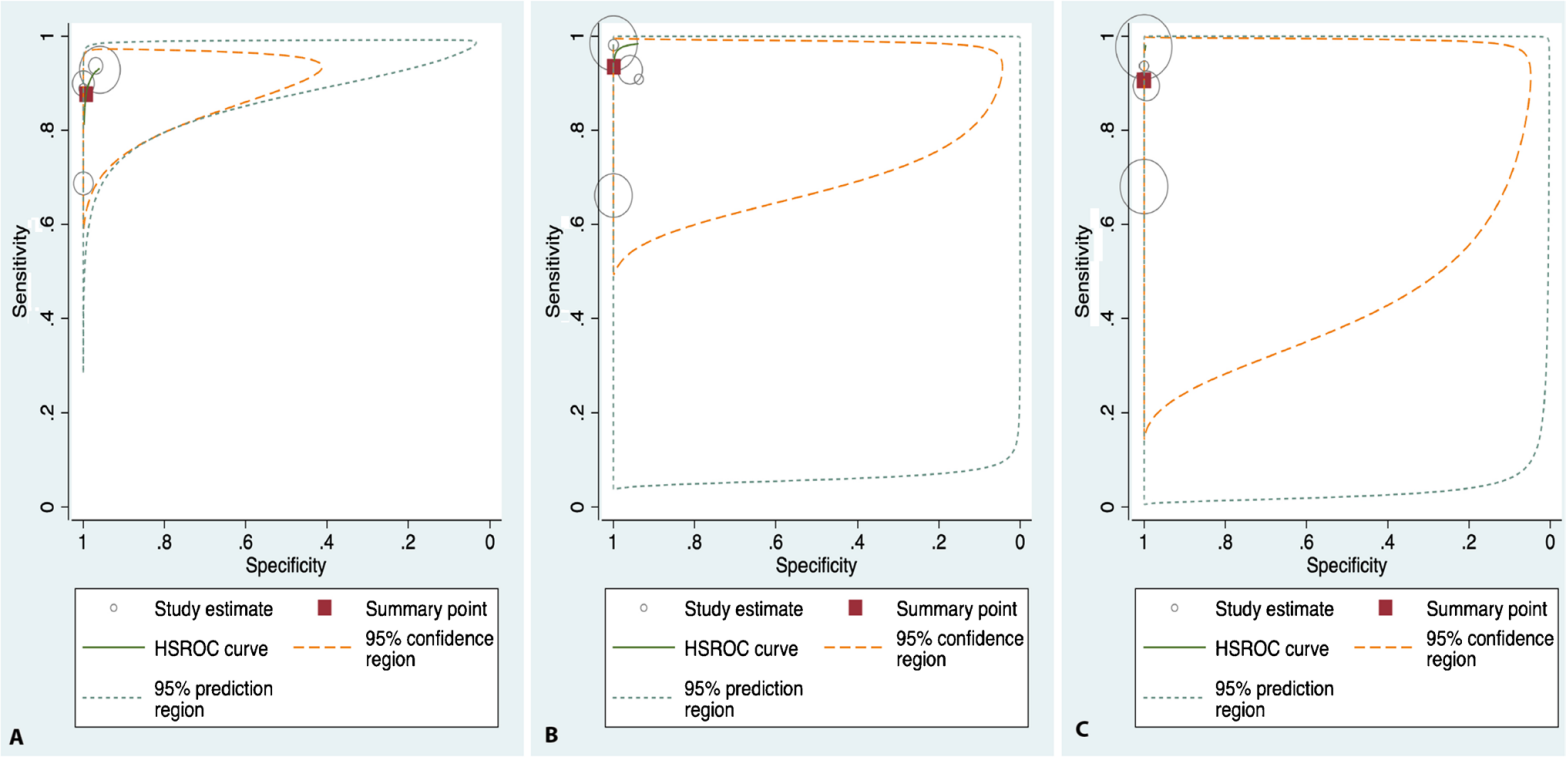
**a** The HSROC curves for CBCT when assessing diagnostic accuracy for scaphoid fractures. **b** The HSROC curves for CBCT when assessing diagnostic accuracy for radiocarpal fractures. **c** The HSROC curves for CBCT when assessing diagnostic accuracy for carpal bone fractures

The sensitivity estimates for CBCT identification of scaphoid fractures ranged from 69 to 94%. The specificity estimates ranged from 96 to 100%. The pooled sensitivity was 87.7% (95% CI 77.6–93.6) and pooled specificity was 99.2% (95% CI 92.6–99.9), as shown in Fig. 5a. The DOR was 940.7 (95% CI 111.3–7951.8). The LR+ and LR− were 116.6 (95% CI 11.7–1157.8) and 0.12 (95% CI 0.06–0.23).

#### Radiocarpal fractures

The sensitivity estimates for CBCT identification of all radiocarpal fractures ranged from 66 to 99%. The specificity estimates ranged from 94 to 100%. The pooled sensitivity was 93.5% (95% CI 81.1–98.0) and pooled specificity was 99.9% (95% CI 91.6–1.00), as shown in Fig. 5b. The DOR was 13,533.9 (95% CI 132.6–1,380,880). The LR+and LR−were 882.2 (95% CI 10.3–75,682.0) and 0.07 (95%CI 0.02–0.20).

#### Carpal fractures

The sensitivity estimates for CBCT identification of carpal fractures ranged from 68 to 98%. The specificity estimate ranged from 99 to 100%. The pooled sensitivity was 90.6% (95% CI 72.7–97.2) and pooled specificity was 100% (95% CI 99–100), as shown in Fig. 5c. The DOR was 24 287.4 (95% CI 635.0–928,866.7). The LR+ and LR− were 2280.0 (95% CI 73.4–7773.6) and 0.09 (95% CI 0.03–0.3).

#### Distal radius fractures

The sensitivity estimates for CBCT identification of distal radius fractures ranged from 65 to 100%. The specificity estimates were 100% in all papers. Reduced data availability limited hierarchical modelling as only 28 distal radius fractures were reported. Pooled values obtained through mixed effects modelling found a sensitivity of 90% (95% CI 67–98) and specificity of 100% (95% CI 10–100).

#### Inter-rater agreement meta-analysis

**Fig. 6:**
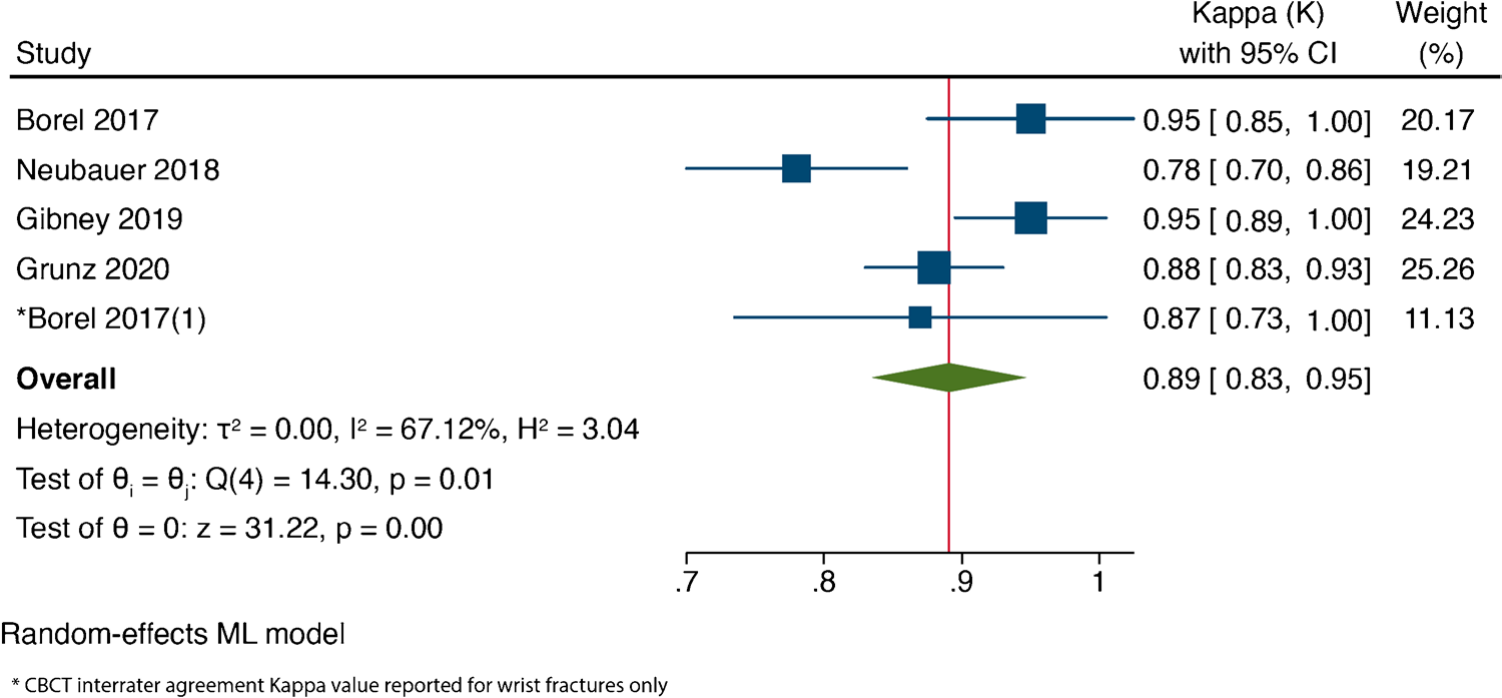
The forest plot of kappa values for reported inter-rater agreement observed in CBCT

Kappa estimates ranged from 0.78 to 0.95. The overall effect for meta-analysis was 0.89 (95% CI 0.82–0.96), as shown in the forest plot in Fig. 6.

## Discussion

The use of flat-panel detectors to perform CBCT image acquisition has recently revolutionised the field of minimally invasive radio-diagnostics. It is increasingly being used for fractures of the extremities, allowing for multiplanar reconstruction. Although a recent metanalysis has been performed on the diagnostic accuracy of CBCT for scaphoid fractures, our study is the first to evaluate its use in acute carpal and distal radius fractures. An excellent diagnostic accuracy of CBCT (AUC 0.99) was seen for all reported radiocarpal fractures [10, 15, 17, 18], with 93.5% sensitivity and 99.9% specificity. Moreover, our results support the use of CBCT in the diagnosis of scaphoid fractures with a pooled sensitivity and specificity of 87% and 99%, respectively, and an overall AUC of 0.98.

Clinical examination in the diagnosis of scaphoid fracture has a wide range of sensitivity (15–100%) and specificity (13–98%). This substantial variation in diagnostic accuracy suggests clinical suspicion is a poor diagnostic indicator of scaphoid fracture if used in isolation [27]. Radiographs are the initial imaging modality of choice but display a relatively poor diagnostic accuracy with a sensitivity between 66 and 81% for scaphoid fractures, 39% for carpal fractures overall and 58% for wrist fractures. Carpal bones have a more complex geometry making them are harder to analyse compared to long bones on radiographs [18]. Radiographs also have a 20–54% false negative rate soon after injury and offer limited information even after 2–6 weeks post-injury. Moreover, it provides unreliable information about fracture alignment, with up to 30–50% of displaced scaphoid fractures missed [26, 28–30]. Thus, the diagnostic value of radiographs in the assessment of radiocarpal fractures is questionable given the varying sensitivity and specificity and poor inter-rater agreement reported by multiple studies [28].

Despite its novelty, there is a very slight difference in inter-rater agreement with CBCT use [18]. Four of the included studies reported substantial or almost perfect inter-rater agreement, as per Landis and Koch agreement scale [31], with excellent confidence as reported by Neubauer et al. [36]. Our analysis demonstrated an almost perfect agreement in the diagnosis of scaphoid fractures using CBCT, with a pooled kappa value of 0.89, irrespective of the radiologist’s experience. In contrast, inter-observer agreement has been shown to be poor for radiographs [24]. This limitation of radiographic assessment is corroborated in the study by Neubauer et al. [19] demonstrating a reduced confidence in reported findings based on radiographs.

In addition to detecting occult fractures, CBCT can aid surgical planning for scaphoid fractures. Grunz et al. [10] showed a change in the management plan for approximately a third of patients based on CBCT findings. Three undetected scaphoid fractures on radiographs underwent surgery based on CBCT findings, whilst five presumed carpal fractures on radiographs were ruled out by CBCT, which would have had unnecessary immobilisation. Similarly, Neubauer et al.’s [19] study reported CBCT imaging resulting in a 15% change in management, with 7% upgraded and 8% downgraded. These findings are comparable to Brink et al. [32] who investigated patients who underwent a single shot MDCT after radiography. In four studies, MRI was performed when there was ongoing clinical concern about occult carpal fractures, which detected additional scaphoid fractures missed on radiography and CBCT scanning [10, 15, 17, 19]. A potential reason for false negative results may be osteopenia, resulting in reduced trabecular and cortical bone thickness.

For scaphoid fractures, conventional computerized tomography (CT) has a sensitivity and specificity of 82% and 96% respectively. CBCT has a comparable diagnostic accuracy to MDCT but with additional benefits [11, 33]. It produces a more detailed image which allows greater visualisation of the area. Spatial resolution which indicates the scanner ability to depict fine detail is an important measure of image quality with regards to bone imaging [14]. CBCT produces sub-millimetre resolution ranging from 0.4 mm to as low as 0.09 mm, whilst standard CT has a spatial resolution of 1–2 mm [34]. Although, to date, no cost–benefit analysis has been performed radiocarpal fractures, Faccioli et al. [35] demonstrated that the introduction of CBCT compared to MDCT in the management of complex finger fractures reduces the time of diagnostic work-up and number of diagnostic procedures, improves quality of life and reduces costs. When trialled in the emergency radiology department, CBCT offered a feasible alternative to MDCT for detection of extremity fractures also increasing patient turnover and reducing radiation exposure [36].

Relative to the annual background radioactivity exposure of 2.4 mSv, and CT effective dose (ED) of 0.03 mSv, CBCT has a very low ED of 0.007 mSv [37]. Grunz et al. [10] used a lower dose for scanning compared to existing literature, with an ED of 4.3 microSv (3.3–5.3), whilst Gibney et al. [15] reported the use of 0.5–1.4 microSv. In comparison, the combined ED for conventional radiography in the assessment for scaphoid injuries has been shown to be up to 2 microSv [38]. Inferentially, CBCT may be performed with an almost comparable ED to radiographs and has an up to 90% lower radiation compared to conventional CT scans [25]. Additionally, it enables the use of lead shielding which is an easy and effective method for further reducing patient exposure to radiation [39]. Lastly, it can be performed with similar positioning of patients for X-ray and thus can be incorporated into a ‘one-stop-shop’ imaging in acute trauma of the extremities [10].

MRI remains the gold standard for wrist fracture diagnoses with a higher sensitivity than CBCT. Nevertheless, our study supports the implementation of CBCT as a reliable examination for the diagnosis of radiocarpal bone fractures, reducing the MRI burden on radiology departments. Diagnosing fractures earlier potentially reduces unnecessary immobilisation, improving cost-effectiveness and reducing patient morbidity.

One of the limitations of CBCT is image acquisition time. The included studies had an acquisition time range of 15–36 s [10, 15, 17–19]. Due to potential positional instability, there is an increased risk of motion artefacts [19]. Whilst Borel et al. [18] suggested stabilisation with straps, Grunz et al. [10] observed no motion artefact in 72.8% of patients with a dedicated extremity machine, and a significantly lower proportion in upper limb extremities compared to lower limb [40]. On the other hand, MRI has a considerably longer acquisition time with image post-processing susceptible to movement artefacts [41].

Additionally, CBCT has been shown to be less effective in diagnosing trabecular bone fractures compared to cortical bone fractures, as it cannot detect soft tissue and bony changes related to such injuries [2]. Nonetheless, trabecular fractures are less common and the clinical implication of this is uncertain as there is no clear consensus on their management [14]. Moreover, CBCT has a limited correlation of bone density with Hounsfield units which may augment image artefacts due to metal implants [12]. Lastly, CBCT has a limited field of vision (FoV) which makes it unsuitable for shoulder and hip fracture assessments, and hence is limited to use in extremities only.

There are several limitations to this meta-analysis. The studies included varying imaging protocols and had a lack of a uniform reference standard. Our studies assumed fractures detected on CBCT to be accurate. Moreover, most studies included patients with ongoing clinical suspicion of scaphoid fractures with negative initial radiography, which may affect the true representativeness of the study population thus limiting the conclusions drawn in this review. Only studies in English were included which may introduce further selection bias. Two of the studies included only occult scaphoid fractures. As occult fractures are more diagnostically challenging, the overall sensitivity and specificity of CBCT might be higher than reported in this study. Given the small number of studies in our review, we were unable to estimate heterogeneity. The use of CBCT for distal upper limb fracture diagnosis is a new initiative and many centres do not have access to CBCT. Further studies with a more robust methodology are required to implement CBCT as a low-dose diagnostic modality for upper limb extremity fractures.

Our study has shown that CBCT is superior to radiographs for radiocarpal cortical fractures. Despite its relatively longer acquisition time, CBCT may be helpful in the diagnostic algorithm as a replacement or supplement (depending on resources available) to radiographs and may improve cost-effectiveness when used in the acute setting. With minimal exposure to radiation, it can obviate the need for unnecessary immobilisation and reduce the burden on the number of follow-up MRI scans required.

## References

[1] Van Onselen E, Karim R, Hage J, Ritt M. Prevalence and distribution of hand fractures. J Hand Surg. 2003;28(5):491–5. https://pubmed.ncbi.nlm.nih.gov/12954264/. 10.1016/s0266-7681(03)00103-7 12954264

[2] Hayat Z, Varacallo M. Scaphoid wrist fracture. StatPearls [Internet]. Treasure Island (FL): StatPearls Publishing 2020 [cited 2021 Jan 15]. Available from: http://www.ncbi.nlm.nih.gov/books/NBK536907/. 30725592

[3] Balci A, Basara I, Çekdemir EY, Tetik F, Aktaş G, Acarer A (2015). Wrist fractures: sensitivity of radiography, prevalence, and patterns in MDCT. Emerg Radiol.

[4] Welling RD, Jacobson JA, Jamadar DA, Chong S, Caoili EM, Jebson PJL (2008). MDCT and radiography of wrist fractures: radiographic sensitivity and fracture patterns. Am J Roentgenol.

[5] Yin Z-G, Zhang J-B, Kan S-L, Wang X-G (2010). Diagnosing suspected scaphoid fractures: a systematic review and meta-analysis. Clin Orthop.

[6] Duckworth AD, Jenkins PJ, Aitken SA, Clement ND, Court-Brown CM, McQueen MM (2012). Scaphoid fracture epidemiology. J Trauma Acute Care Surg.

[7] Clementson M, Björkman A, Thomsen NOB (2020). Acute scaphoid fractures: guidelines for diagnosis and treatment. EFORT Open Rev.

[8] Chunara MH, McLeavy CM, Kesavanarayanan V, Paton D, Ganguly A (2019). Current imaging practice for suspected scaphoid fracture in patients with normal initial radiographs: UK-wide national audit. Clin Radiol.

[9] Carrino JA, Al Muhit A, Zbijewski W, Thawait GK, Stayman JW, Packard N (2014). Dedicated cone-beam CT system for extremity imaging. Radiology.

[10] Grunz J-P, Pennig L, Fieber T, Gietzen CH, Heidenreich JF, Huflage H (2020). Twin robotic x-ray system in small bone and joint trauma: impact of cone-beam computed tomography on treatment decisions. Eur Radiol.

[11] Lang H, Neubauer J, Fritz B, Spira EM, Strube J, Langer M (2016). A retrospective, semi-quantitative image quality analysis of cone beam computed tomography (CBCT) and MSCT in the diagnosis of distal radius fractures. Eur Radiol.

[12] De Smet E, De Praeter G, Verstraete KLA, Wouters K, De Beuckeleer L, Vanhoenacker FMHM (2015). Direct comparison of conventional radiography and cone-beam CT in small bone and joint trauma. Skeletal Radiol.

[13] Ricci M, Boldini M, Bonfante E, Sambugaro E, Vecchini E, Schenal G (2019). Cone-beam computed tomography compared to X-ray in diagnosis of extremities bone fractures: a study of 198 cases. Eur J Radiol Open.

[14] Venkatesh E, Elluru SV (2017). Cone beam computed tomography: basics and applications in dentistry. J Istanb Univ Fac Dent.

[15] Gibney B, Smith M, Moughty A, Kavanagh EC, Hynes D, MacMahon PJ (2019). Incorporating cone-beam CT into the diagnostic algorithm for suspected radiocarpal fractures: a new standard of care?. Am J Roentgenol.

[16] Mallee WH, Walenkamp MMJ, Mulders MAM, Goslings JC, Schep NWL (2020). Detecting scaphoid fractures in wrist injury: a clinical decision rule. Arch Orthop Trauma Surg.

[17] Edlund R, Skorpil M, Lapidus G, Bäcklund J (2016). Cone-beam CT in diagnosis of scaphoid fractures. Skeletal Radiol.

[18] Borel C, Larbi A, Delclaux S, Lapegue F, Chiavassa-Gandois H, Sans N (2017). Diagnostic value of cone beam computed tomography (CBCT) in occult scaphoid and wrist fractures. Eur J Radiol..

[19] Neubauer J, Benndorf M, Ehritt-Braun C, Reising K, Yilmaz T, Klein C (2018). Comparison of the diagnostic accuracy of cone beam computed tomography and radiography for scaphoid fractures. Sci Rep.

[20] Whiting PF, Rutjes AWS, Westwood ME, Mallett S, Deeks JJ, Reitsma JB (2011). QUADAS-2: a revised tool for the quality assessment of diagnostic accuracy studies. Ann Intern Med..

[21] 7.7.7.2 Standard errors from confidence intervals and P values: difference measures [Internet]. [cited 2021 May 16]. Available from: https://handbook-5-1.cochrane.org/chapter_7/7_7_7_2_obtaining_standard_errors_from_confidence_intervals_and.htm.

[22] Deeks JJ, Macaskill P, Irwig L (2005). The performance of tests of publication bias and other sample size effects in systematic reviews of diagnostic test accuracy was assessed. J Clin Epidemiol..

[23] Gibney B, Murphy MC, Ahern DP, Hynes D, MacMahon PJ (2019). Trapezium fracture: a common clinical mimic of scaphoid fracture. Emerg Radiol.

[24] Suojärvi N, Sillat T, Lindfors N, Koskinen SK (2015). Radiographical measurements for distal intra-articular fractures of the radius using plain radiographs and cone beam computed tomography images. Skeletal Radiol.

[25] Pallaver A, Honigmann P (2019). The role of cone-beam computed tomography (CBCT) scan for detection and follow-up of traumatic wrist pathologies. J Hand Surg.

[26] Jørgsholm P, Thomsen N, Besjakov J, Abrahamsson S, Björkman A (2016). MRI shows a high incidence of carpal fractures in children with posttraumatic radial-sided wrist tenderness. Acta Orthop.

[27] Krastman P, Mathijssen NM, Bierma-Zeinstra SMA, Kraan G, Runhaar J (2020). Diagnostic accuracy of history taking, physical examination and imaging for phalangeal, metacarpal and carpal fractures: a systematic review update. BMC Musculoskelet Disord..

[28] Mallee WH, Wang J, Poolman RW, Kloen P, Maas M, de Vet HCW (2015). Computed tomography versus magnetic resonance imaging versus bone scintigraphy for clinically suspected scaphoid fractures in patients with negative plain radiographs. Cochrane Database Syst Rev.

[29] Waeckerle JF (1987). A prospective study identifying the sensitivity of radiographic findings and the efficacy of clinical findings in carpal navicular fractures. Ann Emerg Med..

[30] Bernard SA, Murray PM, Heckman MG (2010). Validity of conventional radiography in determining scaphoid waist fracture displacement. J Orthop Trauma.

[31] Landis JR, Koch GG (1977). The measurement of observer agreement for categorical data. Biometrics.

[32] Brink M, Steenbakkers A, Holla M, de Rooy J, Cornelisse S, Edwards MJ (2019). Single-shot CT after wrist trauma: impact on detection accuracy and treatment of fractures. Skeletal Radiol.

[33] Neubauer J, Benndorf M, Reidelbach C, Krauß T, Lampert F, Zajonc H (2016). Comparison of diagnostic accuracy of radiation dose-equivalent radiography, multidetector computed tomography and cone beam computed tomography for fractures of adult cadaveric wrists. PLoS ONE.

[34] Brüllmann DD, Schmidtmann I, Hornstein S, Schulze RK (2012). Correlation of cone beam computed tomography (CBCT) findings in the maxillary sinus with dental diagnoses: a retrospective cross-sectional study. Clin Oral Investig.

[35] Faccioli N, Santi E, Foti G, Mansueto G, Corain M (2020). Cost-effectiveness of introducing cone-beam computed tomography (CBCT) in the management of complex phalangeal fractures: economic simulation. Musculoskelet Surg.

[36] Jacques T, Morel V, Dartus J, Badr S, Demondion X, Cotten A (2021). Impact of introducing extremity cone-beam CT in an emergency radiology department: a population-based study. Orthop Traumatol Surg Res.

[37] Koskinen SK, Haapamäki VV, Salo J, Lindfors NC, Kortesniemi M, Seppälä L (2013). CT arthrography of the wrist using a novel, mobile, dedicated extremity cone-beam CT (CBCT). Skeletal Radiol.

[38] Behzadi C, Karul M, Henes FO, Laqmani A, Catala-Lehnen P, Lehmann W (2015). Comparison of conventional radiography and MDCT in suspected scaphoid fractures. World J Radiol.

[39] Lee C-H, Ryu JH, Lee Y-H, Yoon K-H (2015). Reduction of radiation exposure by lead curtain shielding in dedicated extremity cone beam CT. Br J Radiol.

[40] Grunz J, Weng A, Gietzen C, Veyhl-Wichmann M, Pennig L, Kunz A, et al. Evaluation of ultra-high-resolution cone-beam CT prototype of twin robotic radiography system for cadaveric wrist imaging. Academic Radiology. 2020. 10.1016/j.acra.2020.06.018. 10.1016/j.acra.2020.06.018 32654956

[41] Havsteen I, Ohlhues A, Madsen KH, Nybing JD, Christensen H, Christensen A (2017). Are movement artifacts in magnetic resonance imaging a real problem?-A narrative review. Front Neurol.

